# Alternative splicing produces structural and functional changes in CUGBP2

**DOI:** 10.1186/1471-2091-13-6

**Published:** 2012-03-20

**Authors:** Hitoshi Suzuki, Makoto Takeuchi, Ayumu Sugiyama, AHM Khurshid Alam, Luyen Thi Vu, Yoshiharu Sekiyama, Hieu Chi Dam, Shin-ya Ohki, Toshifumi Tsukahara

**Affiliations:** 1 Center for Nano Materials and Technology, Japan Advanced Institute of Science and Technology, Ishikawa 923-1292, Japan; 2 School of Materials Science, Japan Advanced Institute of Science and Technology, Ishikawa 923-1292, Japan; 3 School of Knowledge Science, Japan Advanced Institute of Science and Technology, Ishikawa 923-1292, Japan; 4 Japan Science and Technology Agency, ERATO, Shimoda Nano-Liquid Process Project, 2-5-3 Asahidai, Nomi, Ishikawa 923-1211, Japan; 5 Department of Pharmacy, University of Rajshahi, Rajshahi 6205, Bangladesh

## Abstract

**Background:**

CELF/Bruno-like proteins play multiple roles, including the regulation of alternative splicing and translation. These RNA-binding proteins contain two RNA recognition motif (RRM) domains at the N-terminus and another RRM at the C-terminus. CUGBP2 is a member of this family of proteins that possesses several alternatively spliced exons.

**Results:**

The present study investigated the expression of exon 14, which is an alternatively spliced exon and encodes the first half of the third RRM of CUGBP2. The ratio of exon 14 skipping product (*R3δ*) to its inclusion was reduced in neuronal cells induced from P19 cells and in the brain. Although full length CUGBP2 and the CUGBP2 *R3δ *isoforms showed a similar effect on the inclusion of the smooth muscle (SM) exon of the *ACTN1 *gene, these isoforms showed an opposite effect on the skipping of exon 11 in the *insulin receptor *gene. In addition, examination of structural changes in these isoforms by molecular dynamics simulation and NMR spectrometry suggested that the third RRM of R3δ isoform was flexible and did not form an RRM structure.

**Conclusion:**

Our results suggest that CUGBP2 regulates the splicing of *ACTN1 *and *insulin receptor *by different mechanisms. Alternative splicing of *CUGBP2 *exon 14 contributes to the regulation of the splicing of the *insulin receptor*. The present findings specifically show how alternative splicing events that result in three-dimensional structural changes in CUGBP2 can lead to changes in its biological activity.

## Background

The CELF (CUGBP and ETR-3 Like Factor)/Bruno-like protein family plays important roles in the regulation of alternative splicing and translation [1-5]. In mammals, the CELF/Bruno-like family includes six members and is classified into two subgroups based on overall sequence similarity. One group is composed of CUGBP1 and CUGBP2, which share 76% amino acid sequence identity [6]. The other group contains BRUNOL1 (CELF3), BRUNOL5 (CELF5), BRUNOL6 (CELF6), and CELF4, which share 62-66% a.a. sequence identity with each other and 44% sequence identity with CUGBP1 [6]. CELF proteins have two consecutive RNA recognition motifs (RRMs) (RRM1-2) in the N-terminal region and another RRM (RRM3) in the C-terminal region. RRM2 and RRM3 are separated by a linker region that consists of 160-230 amino acids. CELF family members are expressed in multiple tissues with a distinct tissue distribution pattern. CUGBP1 is expressed in almost all tissues, BRUNOL1 and BRUNOL5 are restricted to the brain, and CUGBP2 is abundant in the heart, skeletal muscle, and brain [5,6].

The CELF family protein CUGBP1 was originally identified as an RNA-binding protein for CUG triplet repeats observed in the 3' UTR of the dystrophy myotonica protein kinase (DMPK) mRNA [7]. Increased copies of CUG triplet repeats from 5-37 to 50-5,000 cause myotonic dystrophy type I. CUGBP1 regulates the alternative splicing of exon 5 of cardiac Troponin T (*cTNT*) via the CUG repeats of muscle-specific enhancer elements (MSE) in its pre-mRNAs [1]. CUGBP1 is presumably involved in the incidence of DM because the splicing of *cTNT *was disrupted in DM striated muscle. In addition to its role in the regulation of *cTNT *exon 5 splicing, CUGBP1 is thought to be involved in controlling the alternative splicing of amyloid precursor protein (*APP*) [8], muscle-specific chloride channel [9,10], α-actinin (*ACTN1*) [11,12], and the insulin receptor (*IR*) [13,14]. CUGBP2 (also known as ETR-3, Napor, and Brunol3), a paralogous protein of CUGBP1, is also known as an alternative splicing regulator. Similar to CUGBP1, CUGBP2 activates the inclusion of exon 5 in human *cTNT *mRNA via binding to MSE [3,15]. CUGBP2 induces smooth muscle-specific exon inclusion via binding to uridine purine repeat elements (URE) in *ACTN1 *[11,12]. Although CUGBP2 is known as a splicing activator for the N-methyl-D-aspartate receptor 1 (*NMDA R1*) exon 21, CUGBP2 represses *NMDA R1 *exon 5 inclusion [16]. In addition, CUGBP2 was reported to repress the inclusion of the *IR *exon 11 [17]. These studies showed that CUGBP2 has positive and negative regulatory roles in alternative splicing.

Bruno, another member of this protein family, is a translational repressor involved in germ cell formation in *Drosophila *[2]. The Bruno protein binds to the BRE (Bruno-responsive element) of the 3' UTR of oskar mRNA and represses its translation in the oocyte. The oskar protein is responsible for germ cell formation in the cytoplasm of the posterior pole of the oocyte. In vertebrates, EDEN-BP (embryo deadenylation element-binding protein), an orthologous protein of CUGBP1 in *Xenopus*, has been reported to control the translational regulation activity of specific maternal mRNAs via the EDEN sequence [18]. In addition, not only Bruno-like mRNA but also its protein, which is an orthologous protein of CUGBP1 in zebrafish, localized to the germplasm at the end of the cleavage furrow [11,19,20]. These results suggest that CUGBP1 is involved in translational control and germ cell formation.

The binding sequences of CUGBP2 and CUGBP1 can be classified into two groups. One group is represented by CUG triplet repeats of *cTNT, Mt-PK, DMPK *and *C/EBPβ *[1,7,21], where the binding sequence essentially contains CUG repeats. Another group is represented by the BRE of oskar, EDEN of *Eg5 *and URE of *ACTN1 *[2,11,18], and the binding sequence essentially contains uridine and guanine (UG)-repeats. Although there are three RRMs responsible for RNA-binding in CUGBPs, previous studies concluded that the RRM3 of CUGBPs binds to the UG-repeat in a sequence-specific manner [22]. Contradictory results showed that consecutive RRM1-2s bind to RNA in a sequence-specific manner. In addition to RRMs, the amino acids surrounding the RRMs were reported to affect RNA-binding and splicing activities. Indeed, RRM1-2 plus 70 residues of the adjacent downstream linker and RRM3 plus the last 119 amino acids of the adjacent upstream linker activated the MSE-dependent exon inclusion of *cTNT *[15,17].

Most of the mammalian genes, including *CUGBP2*, are transcribed as alternatively spliced variants. Among the alternative exons of *CUGBP2*, the present study focused on the skipping of exon 14, which encodes the first half of RRM3. Generally, an RRM is composed of two α-helixes and four β-sheets (β1-α1-β2-β3-α2-β4) formed by almost 80 a.a. [23]. The well-conserved RNP-1 and RNP-2 correspond to the third β-sheet (β3) and first β-sheet (β1), respectively. RNP-1 is important for the intercalation of target RNA in a sequence-specific manner [24]. Because exon 14 of the *CUGBP2 *gene encodes the first half of RRM3, a skipping transcript produces CUGBP2 truncated in parts of RRM3 (CUGBP2 R3δ). Similar to CUGBP2 R3δ, there are many proteins that encode unusual or partially truncated RRM domains in the database (Table 1). U2AF35 has an unusual RRM called the U2 homology motif (UHM) and its interaction with U2AF65 strengthens RNA binding [25,26]. Moreover, many proteins that contain partially deleted RRMs appear to be generated by alternative splicing. However, it is unclear how these proteins with partially truncated RRMs caused by alternative splicing function in biological processes.

**Table 1:** List of partial RRMs from the BLAST search

Gene	Isoforms	Total RRMs	Short RRM	Lacking	Tandem	Alternative
CELF4	4	3	3rd	N-ter	*RRM1-2*	Isoform 1/2/3
CPEB1	3	2	2nd	C-ter	RRM1-2	-
EIF3B	1	1	1st	N-ter	-	-
EIF4B	1	1	1st	C-ter	-	-
HNRPAB	2	2	2nd	C-ter	RRM1-2	-
HNRPLL	2	3	1st	C-ter	RRM1-2	-
HTATSF1	1	2	2nd	N- & C-ter	-	-
LEMD3	2	1	1st	N-ter	-	-
LOC100132919	1	1	1st	C-ter	-	-
NONO	2	2	1st	N-ter	RRM1-2	Isoform 1
RBM10	2	2	1st	N-ter	-	Isoform 1
RBM24	2	1	1st	N-ter	-	Isoform 1/3
RBM28	2	3 (4)	1st (2nd)	C-ter	-	Isoform 1
RBM34	2	1 (2)	1st	C-ter	RRM1-2	Isoform 1
RBMS1	2	2	1st	C-ter	RRM1-2	-
RBMS2	1	2	1st	C-ter	RRM1-2	-
RBMS3	1	2	1st	C-ter	RRM1-2	-
RDM1	8	1	1st	N-ter	-	Isoform1/2/3/4
ROD1	3	4	1st	C-ter	-	-
RRP7A	1	1	1st	C-ter	-	-
SRSF1	2	2	1st	C-ter	-	-
SSB	1	2	1st	C-ter	-	-
SYNCRIP	6	3	2nd	C-ter	RRM1-3	Isoform1/2/5/6
TARDBP	1	2	2nd	C-ter	RRM1-2	-
U2AF1	3	1	1st	N-ter	-	-
U2AF2	2	3	3rd	N-ter	*RRM1-2*	-
UHMK1	3	1	1st	N-ter	-	Isoform 3

The list shows human proteins possessing an RRM with a deleted part selected from human RefSeq proteins. In 53 human Among 457 human RRM-type proteins, 53 human RefSeq proteins (27 human genes) showed a severely disrupted RRM domain. When two RRM domains have a short linker region (less than 50 a.a), these RRMs were considered as tandem RRMs. When an alternatively spliced isoform restored the complete RRM, the name of the isoform was listed. The parentheses show RRMs that were removed form the alternatively spliced isoforms.

The present study examined the expression patterns of *CUGBP2 *mRNA and its isoform, *R3δ*, in P19 cells during neural differentiation and in different tissues. *CUGBP2 *was highly expressed in neural cells and in the adult brain compared with *R3δ*, which was the major product in the kidneys, liver and undifferentiated P19 cells. Transient transfection experiments showed similar activities of CUGBP2 and R3δ, and both proteins promoted the use of the SM (smooth muscle) exon instead of the NM (non-muscle) exon of the *ACTN1 *minigene. On the other hand, CUGBP2 and R3δ had opposite effects on alternative splicing of exon 11 of *IR*; CUGBP2 repressed *IR *exon 11 inclusion, whereas R3δ did not and even slightly increased inclusion. This result suggests that the alternatively spliced isoform, R3δ, has a different function from that of CUGBP2. In addition, the results of molecular dynamics (MD) and NMR showed that the structure of the RRM domain differs significantly from that in the R3δ isoform, resulting in the disruption of its binding activity.

## Results and discussion

### Alternative splicing of CUGBP2

**Figure 1:**
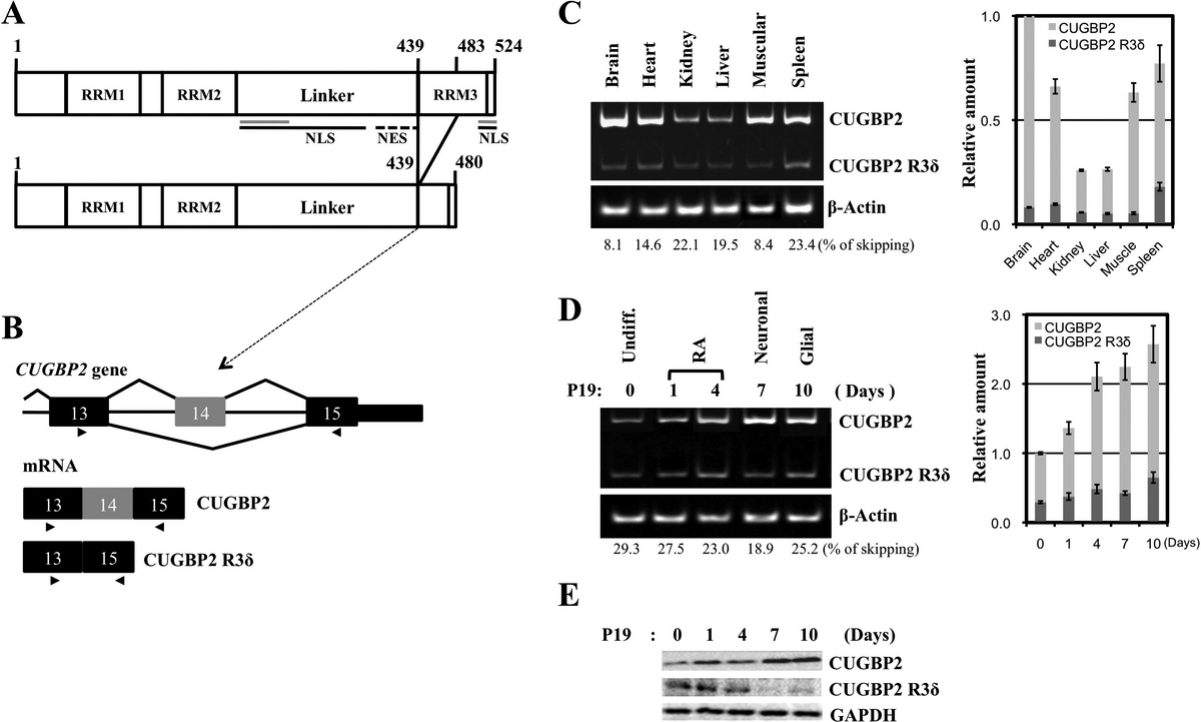
**Alternative splicing of the *CUGBP2 *gene**. (A) Schematic representation of the CUGBP2 protein and the CUGBP2 R3δ isoform. The upper panel shows CUGBP2 and its domains. RRMs represent the RNA-binding domains. The NLS (line), NES (broken line), and splicing activation domain (gray line) were determined in a previous report [27]. The lower panel shows the alternatively spliced form of *CUGBP2*, the *R3δ *isoform. (B) Schematic representation of exon 14 and its adjacent region in the *CUGBP2 *gene. Exons are indicated as black boxes with the alternatively spliced exons indicated as gray boxes. Introns are indicated by a central narrow line. Arrows show primer sites. (C) Expression analysis of *CUGBP2 *in adult mouse tissues. Semi-quantitative RT-PCR was performed using primers to detect the alternatively spliced exon of *CUGBP2*. The right side indicates the positions of exon 14 skipping or inclusion products. *β-Actin *was used as a control. (D) Expression analysis of *CUGBP2 *in P19 neural differentiation. The right side indicates the positions of exon 14 skipping or inclusion products. *β-Actin *was used as a control. Relative amounts of exon 14 skipping and inclusion products were estimated by densitometry. Changes of total expression levels were normalized using brain samples (C) or Day 0 samples (D). The error bars indicate the standard error. The values under the gel images indicate the percentage of the exon 14 skipping in total *CUGBP2 *transcripts. (E) Western blot analysis of CUGBP2. Whole cell extracts of P19 cells (2 μg) were used to detect the changes in the amount of full-length CUGBP2 in the upper panel. The middle panel shows the R3δ isoform detected using 7 μg of each extract. GAPDH was used as a control and is shown in the lower panel.

A search of the UCSC genome browser, BLAT, suggested that the *R3δ *isoform is the product of skipping of exon 14 in 25% of CUGBP2 transcripts. Exon14 is 144 nt and the skipping transcript does not generate a new premature termination codon. To assess in which organ alternative splicing of RRM3 in *CUGBP2 *takes place, RT-PCR was performed in adult mouse tissues. The products of exon 14 inclusion (*CUGBP2*) and exon 14 skipping (*R3δ*), which encode a complete and partial RRM3, respectively, were detected (Figure 1A &1B). *CUGBP2 *mRNA was highly expressed in the brain, where the main product was the exon 14 inclusion transcript encoding a complete RRM3 (Figure 1C, *R3δ *percentage of *CUGBP2 *and *R3δ *in the brain: 8.1%). The total amount of *CUGBP2 *transcripts in the kidney or liver was low compared to that in brain, but the percentage of the *R3δ *isoform was relatively high (Figure 1C, *R3δ *percentage of *CUGBP2 *and *R3δ *in the kidney: 22.1%; in the liver, 19.5%). These results suggest that the *R3δ *isoform is one of major products when their gene expression of *CUGBP2 *is low, and the *CUGBP2 *isoform, but not *R3δ*, is expressed as the major product when their gene expression is high. To further investigate the alternative splicing pattern of *CUGBP2*, RT-PCR was performed in P19 cells during neural differentiation. Increased levels of the exon 14 inclusion product were detected during neural differentiation even though the exon 14 skipping product was not essentially changed (Figure 1D). The *R3δ *percentage on day 7 (neural cell stage) was 18.9%, which was the lowest during P19 neural differentiation (*R3δ *was 29.3% in undifferentiated P19 cells). The relatively low expression of *R3δ *with respect to alternative splicing patterns was also found in tissues of the adult mouse. Western blot analysis of CUGBP2 proteins showed that the full length isoform was the main protein in P19 cells and that its level increased at the neural stage. By contrast, the level of R3δ decreased at the neural stage, although it was observed in undifferentiated P19 cells (Figure 1E).

### Expression analysis of CUGBP2 target genes, *ACTN1 *and *insulin receptor*

**Figure 2:**
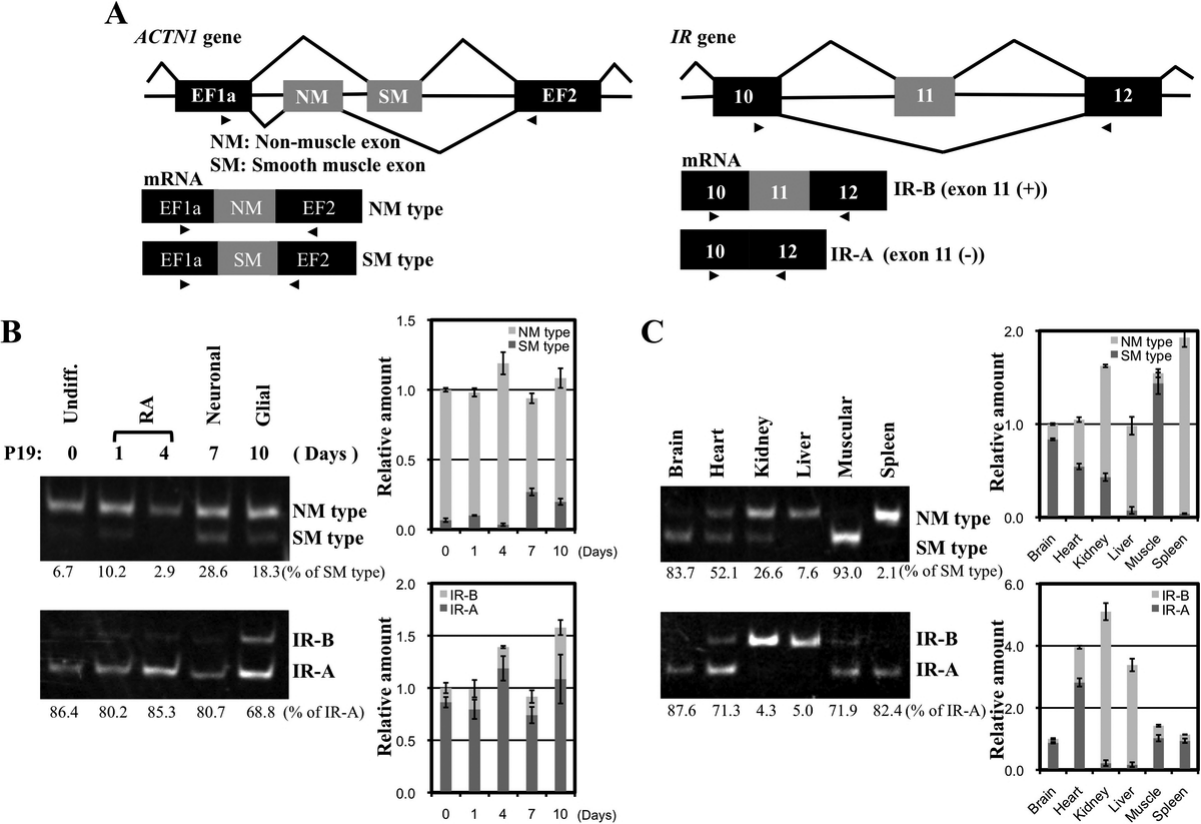
**Expression analysis of the alternative splicing of the *ACTN1 *and *IR *genes**. (A) Schematic representation of the mutually exclusive splicing of the *ACTN1 *and *IR *genes. The genomic structure and alternatively spliced mRNAs of *ACTN1 *are shown in the left panel and those of *IR *in the right panel. Exons are indicated as black boxes with alternatively spliced exons depicted as gray boxes. Introns are indicated with a central narrow line. The arrow indicates the primer sites. (B) Expression analysis of *ACTN1 *and *IR *in P19 neural differentiation. Semi-quantitative RT-PCR was performed using primers to detect the alternatively spliced exons as shown in Figure 2A. The right side shows the positions of the SM exon and NM exon products or exon 11 skipping and inclusion products. (C) Expression analysis of *ACTN1 *and *IR *in adult mouse tissues. Semi-quantitative RT-PCR was performed using primers to detect the alternatively spliced exons. The right side shows the positions of the alternatively spliced products. *β-Actin *is shown as a control in Figure 1. The relative amounts of each PCR product were estimated by densitometry. Total expression levels were normalized using Day 0 samples (B) or brain samples (C). The error bars indicate the standard error. The values under the gel images indicate the percentage of the SM type or IRA in total *ACTN1 *transcripts or *IR *transcripts.

CUGBP2 is a regulator of the alternative splicing of several transcripts, including *ACTN1 *[11] and *IR *[17]. The alternative splicing of *ACTN1 *and *IR *was therefore analyzed in adult mouse tissues and P19 cells. The *ACTN1 *gene has mutually exclusive exons, namely the smooth muscle (SM) exon and non-muscle (NM) exon (Figure 2A). The SM exon., as a percentage of NM and SM exons, was higher in neural differentiated P19 cells (28.6%) than in undifferentiated P19 cells (6.7%, Figure 2B). In the brain, 83.7% of *ACTN1 *transcripts contained the SM exon, while the NM exon was predominant in the kidney (SM exon: 26.6%) and liver (Figure 2C, SM exon: 7.6%). CUGBP2 has been suggested to promote the inclusion of the SM exon in prior work [11,12], suggesting that the elevated expression of *CUGBP2 *and inclusion of the *ACTN1 *SM exon may occur in the same cells and tissues.

On the other hand, the *IR *gene consists of 22 exons and generates 2 isoforms, IR-A and IR-B, which are characterized by the skipping and inclusion of exon 11, respectively (Figure 2A) [28]. RT-PCR analysis of the alternative splicing of the *IR *gene in tissues and P19 cells identified the two isoforms, IR-A and IR-B, in P19 cells during neural differentiation (Figure 2B). Although the expression of the *IR *gene increased during the glial cell stage, the IRA/IR-B ratio did not change significantly during cell differentiation (the IR-A form, as percentage of IR-A and IR-B, was 86.4% in undifferentiated P19 cells and 80.7% in neural differentiated P19 cells). While the exon 11 skipping variant IR-A was expressed in the brain (IR-A: 87.6%), the exon 11 inclusion form IR-B was mainly expressed in the kidney (IR-A: 4.3%) and liver (IR-A: 5.0%). Because CUGBP2 is known to repress the inclusion of *IR *exon 11 [17], elevated expression of *CUGBP2 *and the IR-A variant may occur in the same tissues, but is not observed in P19 cells. In addition to the expression of *CUGBP2*, the ratio of *R3δ *to *CUGBP2 *was lower in the brain than in the kidney and liver. The function of the *R3δ *isoform was therefore examined in detail as described in the next section.

### CUGBP2 R3δ induces smooth muscle type splicing of *ACTN1*

**Figure 3:**
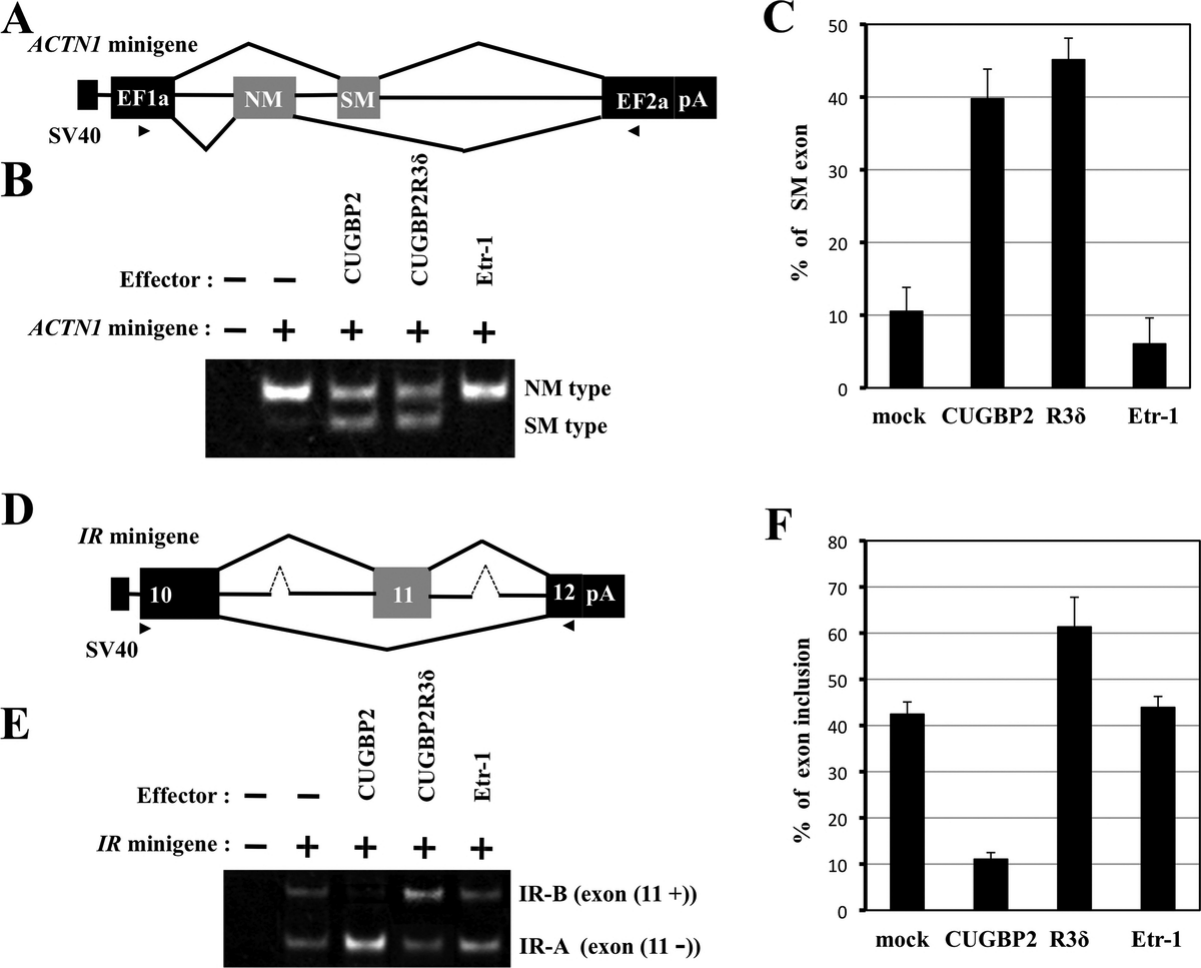
**Transient transfection of the *ACTN1 *minigene and the *IR *minigene**. (A) Schematic representation of the *ACTN1 *minigene. Exons are indicated as black boxes with alternatively spliced exons indicated as gray boxes. Introns are indicated with a central narrow line. The arrows show the primer sites. (B) Results of the transient transfection experiment. COS7 cells were transiently transfected with the *ACTN1 *minigene with/without the CUGBP, CUGBP2 R3δ isoform, or Etr-1 expression vectors. Alternatively spliced products were analyzed by RT-PCR. (C) Densitometric analysis of the transfection products. Quantification of the alternatively spliced products was performed by densitometry. The percentage of SM exon inclusion with respect to total product is shown in graphical representation. The error bars indicate the standard error. (D) Schematic representation of the *IR *minigene. Exons are indicated as black boxes and alternatively spliced exons are indicated as gray boxes. Introns are shown with a central narrow line. The arrows show the primer sites. (E) Results of the transient transfection experiment. HeLa cells were transiently transfected with the *IR *minigene with/without the CUGBP, R3δ isoform, or Etr-1 expression vectors. Alternatively spliced products were analyzed by RT-PCR. (F) Densitometric analysis of the transfection products. Quantification of the alternatively spliced products was performed by densitometry. The percentage of exon 11 inclusion with respect to total product is shown in graphical representation. The error bars indicate the standard error.

As described previously, CUGBP2 promotes the inclusion of the mutually exclusive SM exon instead of the NM exon in *ACTN1 *[11,12]. The specific function of the CUGBP2 isoform R3δ was examined by transient transfection using the mouse *ACTN1 *minigene (Figure 3A). Transfection of COS7 cells with the *ACTN1 *minigene alone resulted in the detection of a transcript including the NM exon as a major product and another transcript with the SM exon as a minor product (Figure 3B). Control COS7 cells lacking the minigene did not show these transcripts, confirming that the two transcripts containing the NM or SM exon were products of the transfected minigene. Co-transfection with a myc-tagged CUGBP2 expression vector resulted in a decrease in the NM exon product concomitant with an increase in the SM exon product (Figure 3B & Additional file 1: Figure S1). Use of the Etr-1 expression vector as a control showed that Etr-1 promoted the use of the NM exon. These results are consistent with previous reports [11].

To assess the function of the alternatively spliced isoform of CUGBP2 R3δ, transient transfection experiments were performed using the *ACTN1 *minigene with the R3δ instead of the CUGBP2 expression vector. R3δ induced the production of the mutually exclusive SM exon similar to CUGBP2 (Figure 3B & S1), suggesting that the function of R3δ in *ACTN1 *splicing is similar to that of CUGBP2 and that part of RRM3, which is encoded in R3δ, may be dispensable for the regulation of the splicing of *ACTN1*. As shown above, our results identified the brain as the location of SM exon inclusion and the kidney and liver as characteristic of NM exon inclusion. In parallel to SM exon inclusion, the expression level of *CUGBP2 *was high in the brain and low in the kidney and liver, suggesting that the upregulation of *CUGBP2 *in the brain contributes to the induction of SM exon inclusion.

### Different effects of CUGBP2 isoforms on the *insulin receptor*

It was reported that CUGBP2 induces the skipping of exon 11 in *IR *[17]. The induction of exon 11 skipping by CUGBP2 was assessed using the *IR *minigene in transient transfection experiments (Figure 3D). Transfection of COS7 or HeLa cells with the *IR *minigene alone resulted in the production of similar amounts of exon 11 inclusion and skipping products (Figure 3E). Exon 11 inclusion and skipping products were not detected in COS7 or HeLa cells without the minigene, confirming that they were transcribed from the transfected minigene. Co-transfection of the CUGBP2 expression vector and the *IR *minigene caused an increase in the exon 11 skipping product and a decrease in the exon 11 inclusion product (Figure 3E & S1). Co-transfection of Etr-1 as a control did not cause significant changes compared with the transfection of the minigene alone (Figure 3E &3F). These results confirmed that CUGBP2 induces the skipping of exon 11, as previously reported. This effect was clearer in HeLa cells than in COS7 cells (Figure 3E, data not shown).

To examine potential changes in the function of the alternatively spliced isoform, R3δ, the *IR *minigene was co-transfected with the R3δ expression vector, which caused a decrease in exon 11 skipping and an increase in exon 11 inclusion in comparison to co-transfection with CUGBP2 or the mock control (Figure 3E &3F). In a previous report, artificially truncated CUGBP2 proteins with deleted N-terminal regions or deleted C-terminal regions were unable to engage in efficient exon 11 skipping [17]. In the present study, R3δ promoted the exon 11 inclusion, which is in contrast to the activity to full-length CUGBP2, which promotes exon 11 exclusion of the *IR *gene. These results also indicate that part of RRM3, which is encoded by the alternative exon (exon 14) of *CUGBP2*, is not only essential for the skipping of *IR *exon 11, but that disruption of this part of RRM3 may result in a change in the splicing regulated by CUGBP2. Among CELF family proteins, an isoform resembling R3δ can be predicted in CELF4 (Table 1). However, a generalized rule for RRM3s cannot be established because exon 11 skipping can be activated by a subgroup of proteins that includes CUGBP1 and 2, but not by another subgroup that includes Etr-1 (CELF3) or CELF4 (Figure 3E) [17,29]. Our results suggest that the disruption of part of RRM3 in CUGBP2 generates a new activity in the regulation of the splicing of the *IR *gene. Because the R3δ isoform has a similar activity to CUGBP2 in the regulation of splicing of *ACTN1*, it cannot be considered simply as a dominant negative isoform of CUGBP2. The product of exon 11 skipping was detected in the brain and the product of exon 11 inclusion was detected in the kidney and liver, suggesting that high CUGBP2 expression in the brain contributes to exon 11 skipping. A high ratio of *R3δ *to *CUGBP2 *in the kidney and liver may repress exon 11 skipping due to the specific function of the R3δ isoform.

### Structural analyses of the third RRM of the CUGBP2 R3δ isoform

A total of 457 human RefSeq proteins with RRM domain(s) were found in the BLAST. An RRM domain is usually 70 ~ 80 a.a. in length, and slightly smaller RRM domains (~ 60 a.a), which appear to lack rnp-2 in the N-terminus or the dimerization module in the C-terminal region, are frequently observed in conserved domain searches of BLAST. RRM3 of CUGBP2 consists of 75 a.a., and 44 a.a. of this region are disrupted in the R3δ isoform. Disruption of almost half of RRM did not prevent recognition of the remaining sequence in the conserved domain search. A total of 53 human RefSeq proteins (27 human genes) with severely disrupted RRM domains are listed in Table 1. Some of the domains shown in Table 1 such as eIF3B, eIF4B, La (SSB), U2AF35 (U2AF1) and U2AF65 (U2AF65), have been reported to form unusual RRM structures, although the amino acid sequences were not sufficient to predict specific RRM structures [26,30-32].

**Figure 4:**
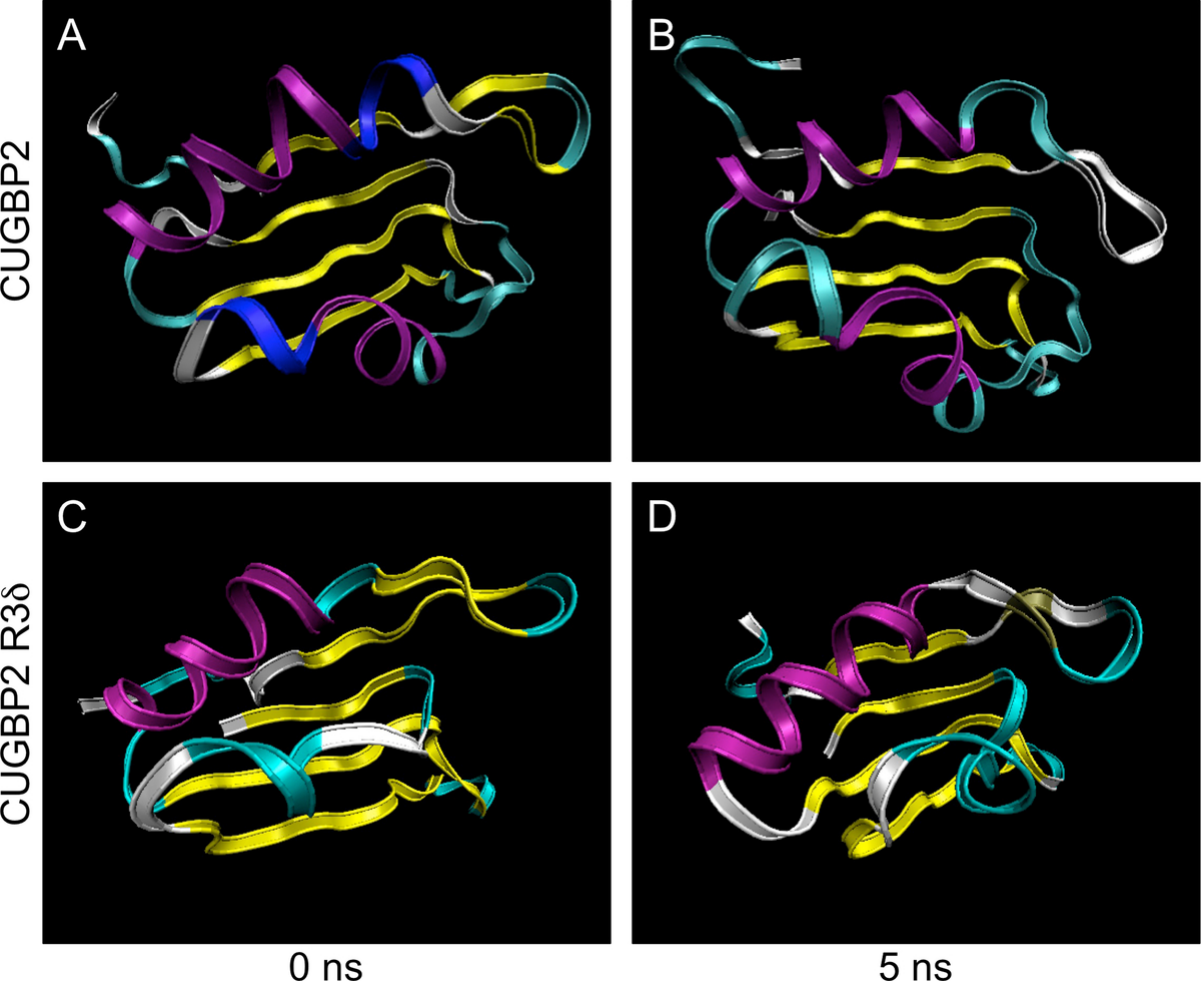
**Structure transitions of the CUGBP2 and R3δ isoforms**. (A) Initial structure of the CUGBP2 RRM3. The structure was predicted using CUGBP1 RRM3 (2rq4a) because the amino acid sequences of RRM3 of CUGBP1 and 2 are almost identical. (B) MD simulation of the CUGBP2 RRM3. MD simulation was performed for 5 ns. (C) Initial structure of the R3δ isoform RRM3. The RRM3 and the linker residues of the R3δ isoform were analyzed by comparative modeling by SFAS in PBDj. (D) MD simulation of the R3δ isoform. The result of comparative modeling was used for MD simulation for 5 ns to analyze the folding and stability of the predicted structure.

**Figure 5:**
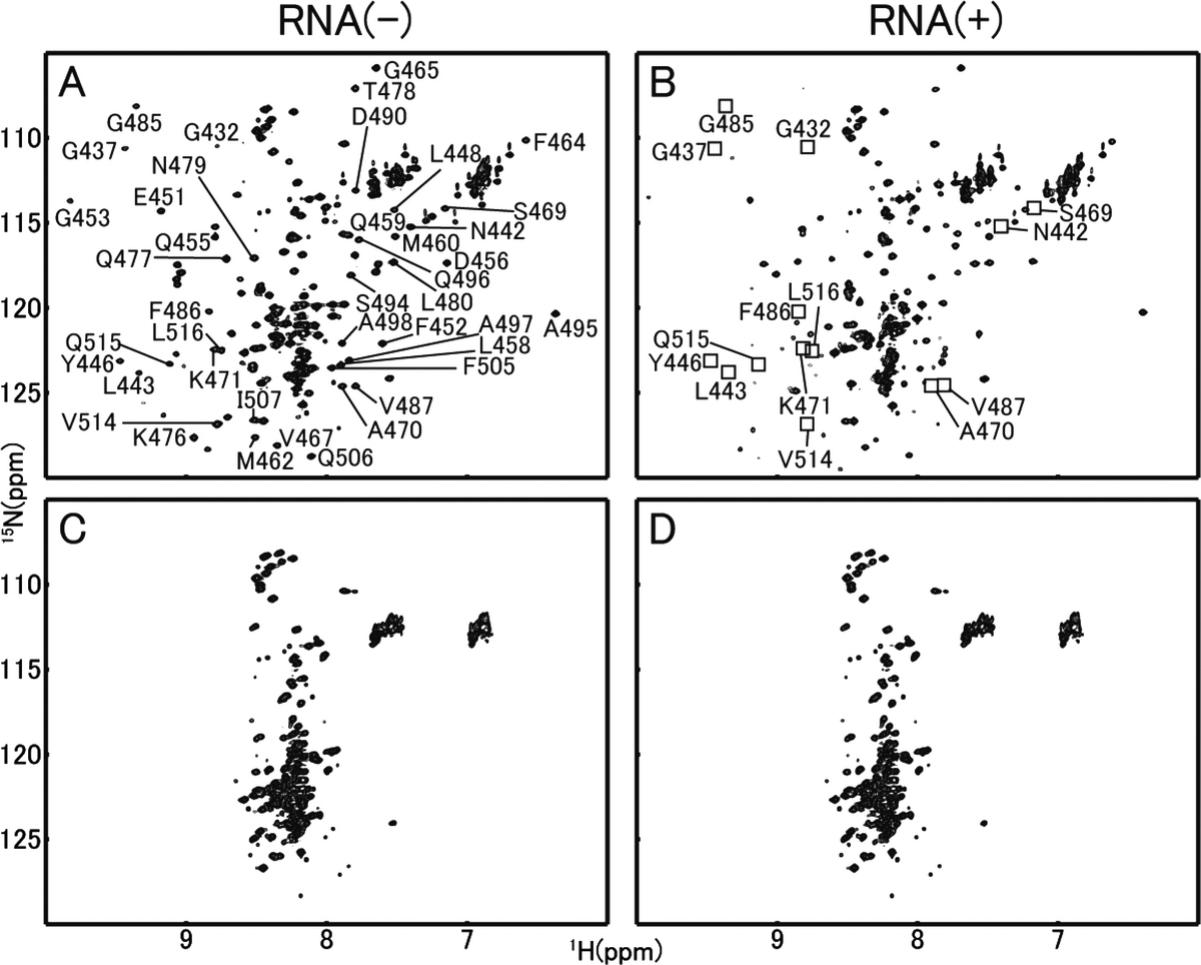
**^1^H-^15^N HSQC spectra of CUGBP2 and the R3δ isoform**. The ^1^H-^15^N HSQC spectra of CUGBP2 and the R3δ isoform in the absence (left) and presence of RNA (protein:RNA = 1:1, right). For clarity, only certain resonances are labeled with one-letter amino acid codes and numbers [for further details, see Tsuda *et al.*, 2009] [22]. The open boxes of panel B highlight the resonances that disappear with the addition of (UG)3 RNA

There-dimensional structures of the RRM3 isoforms were examined by MD simulation and NMR spectroscopy. The structural analysis of the RRM3 of CUGBP1 has been reported previously [22]. Several unaligned residues were found between the RRM3s of CUGBP1, and 2. The results of homology modeling showed that the third domain of CUGBP2 formed an RRM structure (Figure 4A). No significant difference could be observed between the MD simulations of RRM3 of CUGBP1 and RRM3 of CUGBP2. (data not shown). Further, CUGBP2 RRM3 maintained an RRM structure for 5 ns in the MD simulation (Figure 4B). The ^1^H-^15^N HSQC spectrum of the RRM3 of CUGBP2 in the absence of RNA showed that, unlike RRM3 of CUGBP1, the resonances of the N-terminal residues in the linker domain were concentrated in the center of the spectrum, suggesting a random coil structure. This result was probably due to the inclusion of a long N-terminal sequence in the present constructs, which forms a flexible linker region connecting RRM2 with RRM3 (Figure 5A).

Protein threading and comparative modeling of the R3δ isoform were performed in the PDBj. The RRM3 of human CUGBP1 was chosen as the ideal template in the search and it was predicted that the R3δ isoform with part of the linker sequence would not form an RRM structure, especially as the first α-helix (α1) was missing (Figure 4C). We used MD simulations to investigate whether the addition of linker residues to the RRM domain could restore its structure. However, even in these conditions, the first α-helix cannot be formed even after 5 ns (Figure 4D). Moreover, resonances corresponding to the R3δ isoform were detected in the central region of the NMR spectrum, suggesting the presence of greater alterations in the structure of R3δ than those shown by MD simulation (Figure 5C) and indicating that the RRM structure cannot be restored in the R3δ isoform. In addition, the interaction between CUGBP2 or the R3δ isoform and RNA molecules was examined. The addition of RNA caused the disappearance of many resonances and the appearance of new resonances in CUGBP2 (Figure 5B). The affected residues, which are highlighted with an open box in Figure 5B, were consistent with the corresponding region of CUGBP1 [22] and indicate that CUGBP2 binds to (UG)_3 _in a similar manner as CUGBP1. No spectral changes were detected in the R3δ isoform with the addition of RNA (Figure 5D), indicating that the R3δ isoform does not bind to (UG)_3_.

Although the RNA-binding of RRM1-2 of CUGBPs is still unclear, RRM3 is known to be responsible for RNA-binding to UG-repeats [22]. MD simulation suggested that the R3δ isoform could not form an RRM domain and the NMR spectrum showed that it did not bind to the UG-repeat. These results lead to the speculation that the disruption of the first half of RRM3 by exon 14 skipping causes a defect in RNA-binding. However, the R3δ isoform may interact with *ACTN1 *pre-mRNA based on the fact that this isoform still has two RRMs and two different *cis*-elements were reported in the alternative splicing of *ACTN1 *[11,12]. A CUGBP1 interacting *cis*-element was determined in the intronic region of *IR *and shown to affect the alternative splicing of *IR *[33]. Because this sequence is not a typical UG-repeat, perhaps the RRM3 may not be responsible for the primary interaction with the *IR *pre-mRNA. Therefore, other, currently unknown, proteins might interact with the RRM3 domain and it might be the interactions of these proteins rather than RRM3 RNA-binding activity that is abrogated by RRM3 alternative splicing.

## Conclusion

The results of the present study show that while CUGBP2 and its R3δ isoform similarly promoted the use of the SM exon instead of the NM exon in the alternative splicing of the *ACTN1 *minigene, they did not similarly promote the use of exon 11 in the alternative splicing of the *IR *minigene; CUGBP2 promoted skipping of exon 11, whereas R3δ did not. In addition, the results of MD simulation and NMR suggested that the truncated RRM3 region in R3δ resulting from the alternative splicing of CUGBP2 could neither form a new RRM domain nor bind to a UG-repeat. Thus, it is possible that other, currently unknown, CUGBP2-interacting proteins and/or other splicing factors involved in splicing regulation might be important for RRM3 function. Our results also showed that the skipping of exon 14 of *CUGBP2 *not only disrupted CUGBP2 RNA-binding activity but also altered its splicing regulator function. A high ratio of *R3δ *to *CUGBP2 *in the kidney and liver may affect the splicing of the *IR *gene and repress exon 11 skipping.

## Methods

### Plasmid construction

The entire open reading frame of *CUGBP2 *cDNA (NM_001110228.1) and its alternatively spliced isoform, *CUGBP2 R3δ *(NM_010160.2), were amplified by RT-PCR using mouse brain total RNA and the following primers: TGTACTCGAGATGCGCTGTCCCAAATCC and TCTGTCTAGAGGATCAGTAAGGTTTGCTGTCG. The resulting cDNAs were subcloned into the pCS2+ MT vector using Xho I and Xba I sites. RRM3 and RRM3 R3δ were amplified by RT-PCR using the primers GCTCGGATCCATGGCGGCTCTGAATGG and TCTGCTCGAGGATCAGTAAGGTTTGCTGTCG, and were subcloned into the pGEX6P-1 vector using Bam HI and Xho I sites. The coding sequence of Etr-1 was amplified by RT-PCR using mouse brain total RNA and subcloned into the pCS2+ MT vector. The preparation of the *ACTN1 *minigene was described previously [34].

### P19 cell culture and cell differentiation

Embryonic carcinoma P19 cells were cultured as described elsewhere [35]. For neural differentiation, P19 cells were allowed to aggregate in Petri dishes (Falcon) at a seeding density of 1 × 10^5 ^cells/ml in the presence of 1 μM all-trans-retinoic acid (RA, Sigma) in α-MEM (Minimum Essential Medium, Sigma) supplemented with 10% FBS (Fetal Bovine Serum, Sigma). After 4 days of aggregation, cells were dissociated into single cells by 0.25% trypsin-ethylenediaminetetraacetic acid (EDTA) (Sigma) solution and replated in a tissue culture dish at a density of 3-6 × 10^5 ^cells/ml. The cells were then allowed to adhere and were cultured in the absence of RA for 10 days. Media were replaced every 48 hours. COS7 and HeLa cells were grown in DMEM (Dulbecco's Modified Eagle's Medium) supplemented with 10% FBS (Fetal Bovine Serum, Hyclone).

### RNA purification and semi-quantitative RT-PCR

Purification of total RNA from P19 cells was performed using the TRIzol reagent (Invitrogen) after the cells were washed three times with ice-cold PBS. Total RNAs from adult mouse tissues were commercially available (Ambion, Toyobo). cDNA synthesis was performed by SuperScript III (Invitrogen) with oligo-dT primers, using 1 μg of total RNA in 20 μl reaction mixture. PCR reactions were carried out in 20 μl of reaction mixture containing 1 μl of template cDNA, 0.1 U of Go Taq Flexi DNA polymerase (Promega), 1 × GO Taq Flexi Buffer, 2.5 mM MgCl_2_, 0.2 mM dNTPs and 4 pmol of each primer. The PCR conditions consisted of an initial denaturation step at 95°C for 3 min, followed by a cycle of denaturing at 95°C for 30 s, annealing at 60°C for 30 s, and extension at 72°C for 30 s. The primer names, sequences and number of cycles were as follows: CUGBP2 exon 13, CACTGCCCACTTTGTACAGC, and CUGBP2 exon 15, CTGATCCTAACCCCAGAAGC, with 30 cycles; ACTN1 exon EF1a, CGCCTCTTTCAACCACTTTG, and ACTN1 exon EF2, TCATGATTCGGGCAAACTCT with 27 cycles; and IR exon 10, CCTTCGAGGATTACCTGCAC, and IR exon 12, TGTGCTCCTCCTGACTTGTG, with 32 cycles. *β-Actin *was used as an internal control as described previously [34]. PCR products were analyzed in 6% polyacrylamide gels stained with Ethidium Bromide (EtBr) and visualized in an UV-trans illuminator (Vilber Lourmat). Each RT-PCR experiment was performed more than three times. Quantitative densitometry of the bands was performed using Image Gauge software (Fuji Film).

### Western blot analysis

Western blotting was performed according to a previously described experimental protocol [36]. Cultured P19 cells were washed with ice-cold PBS, collected in 1× buffer D, and sonicated 3 times for 30 s on ice. One microgram and 2 μg of whole cell lysates were analyzed by 8% SDS-polyacrylamide gel electrophoresis to detect full-length GAPDH and CUGBP2, respectively. Because the expression level of the R3δ isoform was low, 7 μg of lysate was used in one lane. The positions of the full-length (56 kDa) and R3δ (51 kDa) isoform were determined with molecular weight markers (Bio-rad). Anti-CUGBP2 (GENWAY, 1:1000) and anti-GAPDH (IMGENEX, 1:1000) were used as primary antibodies. Anti-rabbit IgG-HRP (GE, 1:2000) conjugated antibody was used as the secondary antibody to detect CUGBP2 and R3δ proteins. Anti-goat IgG-HRP conjugated antibody (Invitrogen, 1:3000) was used as secondary antibody to detect GAPDH. The experiments shown in Additional file 1: Figure S1 were performed with 1 μg of whole cell extracts and anti-myc (CST, 1:1,000) as a primary antibody and anti-rabbit IgG HRP conjugated antibody as a secondary antibody. The membranes were treated using the ECL kit (GE), and the images were analyzed using the LAS-3000 analyzer (Fuji Film).

### Transient transfection experiments

Transient transfection experiments were carried out as described previously [36]. Transfection was performed using Lipofectamine 2000 (Invitrogen) according to the manufacturer's instructions. Total RNAs were isolated from transfected cells using the TRIzol reagent (Invitrogen). cDNA synthesis was performed in Superscript III (Invitrogen) with the T7 primer. PCR was performed as described above using the IR exon 10 primer CCTGAAGGAGCTGGAGGAG and the IR exon 12 primer ACCGTCACATTCCCAACATC with 25 cycles, and the primers of ACTN1 exon EF1a and ACTN1 exon EF2 with 25 cycles. The PCR products were analyzed in 6% native polyacrylamide gels stained with EtBr. The images were then visualized in an UV-transilluminator (Vilbar lourman). Each transfection experiment was performed more than three times. Quantitative densitometry of the bands was performed using Image Gauge software (Fuji Film).

### Protein expression

Fragments of CUGBP2 and the R3δ isoform were expressed in *Escherichia coli *strain BL21(DE3) using the pGEX-6P-1 expression plasmid. The two constructs for GST-CUGBP2 and the GST-R3δ isoform were grown in M9 minimal medium containing 1 g/L [^15^N] ammonium chloride (Cambridge Isotope Laboratories) as the sole source of nitrogen until an OD600 of 0.6 was reached, followed by induction with 0.1 mM IPTG. The cultured GST-CUGBP2 and GST-R3δ cells were allowed to grow for an additional 8 hours at 30°C and 6 hours at 25°C, respectively. The cells were harvested by centrifugation at 4,000 rpm for 20 min. The harvested cell pellets of CUGBP2 and the R3δ isoform were resuspended in buffer 1 [50 mM Tris-HCl (pH 8.0) and 1 mM DTT] and buffer 2 [20 mM Tris-HCl (pH 8.0), 150 mM NaCl, 50 mM glycine ethyl ester, 25 mM arginine, 25 mM glutamic acid and 10 mM DTT], respectively. The cell solutions were lysed by sonication and the lysate was clarified by centrifugation at 15,000 rpm for 20 min followed by filtration with a 0.45 μm membrane.

### Purification of CUGBP2 and the R3δ isoform

Protein lysates were loaded onto a Q-Sepharose column and eluted with a stepwise concentration gradient of 100-300 mM NaCl in 20 mM Tris (pH 8.0) buffer. Fractions containing GST-tagged CUGBP2 were identified using SDS-PAGE. A HiLoad 16/60 Superdex 75 column (GE Healthcare) connected to an AKTA prime plus (GE Healthcare) was equilibrated with 20 mM Tris-HCl buffer (pH 8.0) containing 150 mM NaCl and 1 mM DTT with a flow rate of 1.0 mL/min. The sample was concentrated and applied to the column.

Fractions containing GST-tagged CUGBP2 were identified using SDS-PAGE and pooled. CUGBP2 was cleaved from the GST-tagged protein by 40 units of PreScission protease and extensively dialyzed against PreScission protease cleavage buffer [50 mM Tris-HCl (pH 7.0), 150 mM NaCl, 1 mM EDTA and 1 mM DTT] at 4°C for 48 hours. The cleaved proteins were again applied to a gel filtration column. Finally, CUGBP2 was applied to a GST column, and the flow-through fractions were collected. The pure protein solution was dialyzed extensively against a 20 mM NH4HCO3 solution and then lyophilized.

The GST-tagged R3δ isoform was isolated from sonicated cell extracts with a glutathione Sepharose 4 Fast Flow (GE Healthcare) column according to a standard protocol. The R3δ isoform was cleaved from the fusion protein by PreScission protease and the GST-tags were removed with a GST column under the same conditions as those used for CUGBP2. Finally, the R3δ isoform was purified by a reverse phase HPLC COSMOSIL C18 column (10 mm I.D. × 250 mm, Nacalai Tesque Inc.) protected by a guard column. Eluent A was 100% water with 0.1% (v/v) trifluoroacetic acid (TFA); eluent B was 99.8% (v/v) acetonitrile with 0.1% (v/v) TFA. The gradient was as follows: 0 min, 5% B; 5 min, 5% B; 70 min, 70% B. The flow rate was 2 mL/min. The UV wavelength was 280 nm. Protein fractions from 50-51 min were collected, dialyzed and then lyophilized.

### Nuclear magnetic resonance (NMR) spectroscopy

For NMR measurements, the samples were concentrated to 0.1 mM in 20 mM Bis-Tris (pH 7.0) containing 100 mM NaCl, 1 mM 1,4-*DL*-dithiothreitol-*d*10 (*d*-DTT) and 0.02% NaN_3 _(in 90% H_2_O/10% D_2_O), using an Amicon Ultra-4 filter (3000 MWCO, Millipore). The NMR experiments were performed for the RNA-free forms and for the RNA-bound forms on an 800 MHz spectrometer (Bruker AVANCE III 800) equipped with a TCI-cryogenic probe at 15°C. The NMR data were processed using NMRPipe [37]. Analyses of the processed data were performed with the program NMRViewJ [38]. The ^15^N chemical shift was calculated by using the ratio, γ_N_/γ_H _= 0.101329118.

### Comparative modeling and molecular dynamics simulation

Comparative modeling was performed in the PBDj site http://sysimm.ifrec.osaka-u.ac.jp/sfas/. MD simulations were carried out to confirm the structural stability of the RRM3 conformation in CUGBP2 and CUGBP2 R3δ. Detailed information on these proteins and the MD conditions used is provided in Table 2. The pdb files of prediction and simulation are available as Additional file: Additional file 2A is the pdb for the RRM3 of CUGBP2 at 0 ns, Additional file 3: 2B shows the pdb for CUGBP2 at 5 ns, Additional file 4: 2C is the pdb for R3δ at 0 ns, and Additional file 5: 2D corresponds to the pdb for R3δ at 5ns. MD simulations were carried out by using the AMBER10 program package with force field 03 [39]. Simulation time was 5 ns (Δt = 1fs) at room temperature (300K) with periodic boundary conditions.

**Table 2:** The conditions of molecular dynamic simulations

	CUGBP2	CUGBP2 R3δ
# Total atoms	39883	28754
# Protein atoms	1402	1131
# Sol water	12821	9205
# Ions	3	4

## Authors' contributions

HS carried out the design of the study, helped to perform the transient transfection assay, and drafted the manuscript. MT carried out the NMR experiment and helped to draft the manuscript. AS carried out the MD study and helped to draft the manuscript. AKA carried out expression analysis and transient transfection assays. LTV participated in expression analysis and transient transfection assays. YS carried out plasmid construction for the NMR experiment and transient transfection assays. HCD participated in the MD study. SO participated in the NMR experiment and helped to the draft manuscript. TT participated in the design of the study and helped to draft the manuscript. All authors read and approved the final manuscript.

## Supplementary Material

Additional file 1 **Figure S1**. Transient transfection of the effectors with the *ACTN1 *minigene or the *IR *minigene. Transient transfection experiments with the *ACTN1 *minigene (upper panel) or the *IR *minigene (lower panel) were performed as shown in Figure 3. Whole cell extracts were analyzed by western blot analysis using an anti-Myc or anti-GAPDH antibody. Endogenous expression of GAPDH and over-expressing effectors containing CUGBP2, R3δ, and Etr-1 were observed. Click here for file

Additional file 2 **Additional file 1A**. The pdb file of the predicted CUGBP2 RRM3 at 0 ns. Click here for file

Additional file 3 **Additional file 1B**. The pdb file of the simulated CUGBP2 RRM3 at 5 ns. Click here for file

Additional file 4 **Additional file 1C**. The pdb file of the predicted structure, which contains the partial RRM3 and linker region in CUGBP2 R3δ, at 0 ns. Click here for file

Additional file 5 **Additional file 1D**. The pdb file of the simulated structure, which contains the partial RRM3 and linker region in CUGBP2 R3δ, at 5 ns. Click here for file
