# Supplementary figures and images for: Alternative splicing produces structural and functional changes in CUGBP2
Source: BMC Biochem. 2012 Mar 20;13:6. doi: 10.1186/1471-2091-13-6 (PMC3368720; doi:10.1186/1471-2091-13-6)

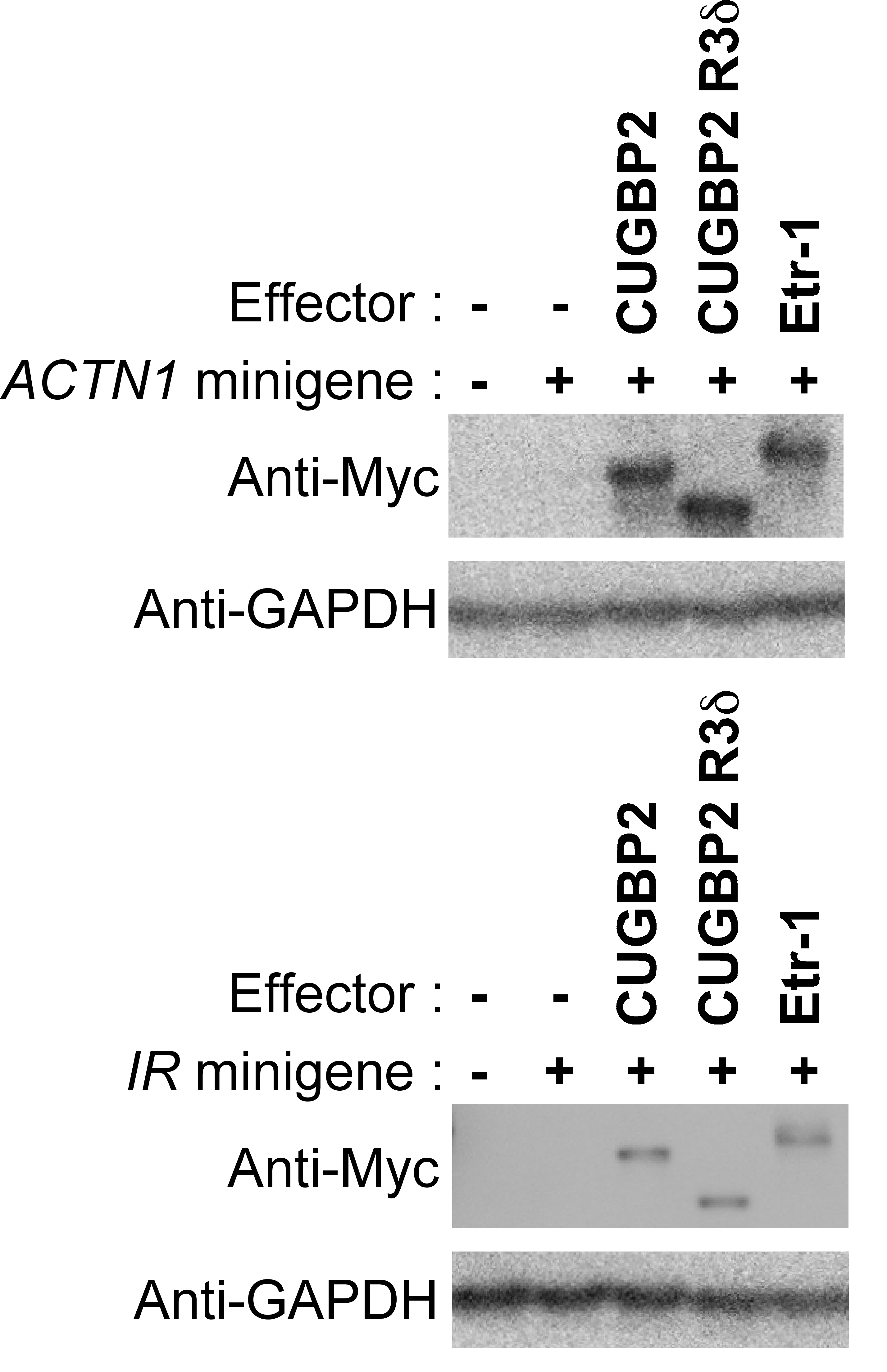

Supplement: Additional file 1 — Figure S1. Transient transfection of the effectors with the ACTN1 minigene or the IR minigene. Transient transfection experiments with the ACTN1 minigene (upper panel) or the IR minigene (lower panel) were performed as shown in Figure 3. Whole cell extracts were analyzed by western blot analysis using an anti-Myc or anti-GAPDH antibody. Endogenous expression of GAPDH and over-expressing effectors containing CUGBP2, R3δ, and Etr-1 were observed. [file 1471-2091-13-6-S1.PNG]
